# Supplementary material for: A standardised differential privacy framework for epidemiological modeling with mobile phone data
Source: PLOS Digit Health. 2023 Oct 27;2(10):e0000233. doi: 10.1371/journal.pdig.0000233 (PMC10610440; doi:10.1371/journal.pdig.0000233)
Supplement: S1 Fig — Scenarios 1 and 2 the disease is spread in two large and small counties, respectively; Scenarios 3, 4, and 5 the epidemic occurred in counties with no, medium to high connectivity with neighboring counties; Scenarios 6, 7, and 8 key parameters such as the burden of asymptomatic, the contact rate varied. Is, Ia, and Obs are infected symptomatic, infected asymptomatic, and observed. (DOCX) [file pdig.0000233.s001.docx]

**A standardised differential privacy framework for epidemiological modeling with mobile phone data**

Merveille Koissi Savi^1^, Akash Yadav^2^, Wanrong Zhang^3^, Navin Vembar^4^, Andrew Schroeder^2^, Satchit Balsari^5^, Caroline O. Buckee^6^, Salil Vadhan^3^, Nishant Kishore^6*^

^1^Department of Medical Oncology, Dana Farber Cancer Institute, Harvard School of Medicine, Boston, Massachusetts, United States of America

^2^ Direct Relief, Santa Barbara, California, United States of America

^3^ Department of Computer Sciences, Harvard John A. Paulson School of Engineering & Applied Sciences, Boston, Massachusetts, United States of America

^4^ Camber Systems, Washington, District of Columbia, United States of America

^5^ Department of Emergency Medicine, Harvard Medical School, Boston, Massachusetts, United States of America

^6^Department of Epidemiology, Harvard TH Chan School of Public Health, Boston, Massachusetts, United States of America

*Corresponding author: [nish.kishore@gmail.com](mailto:koissi_savi@dfci.harvard.edu)

**S1 Fig.**


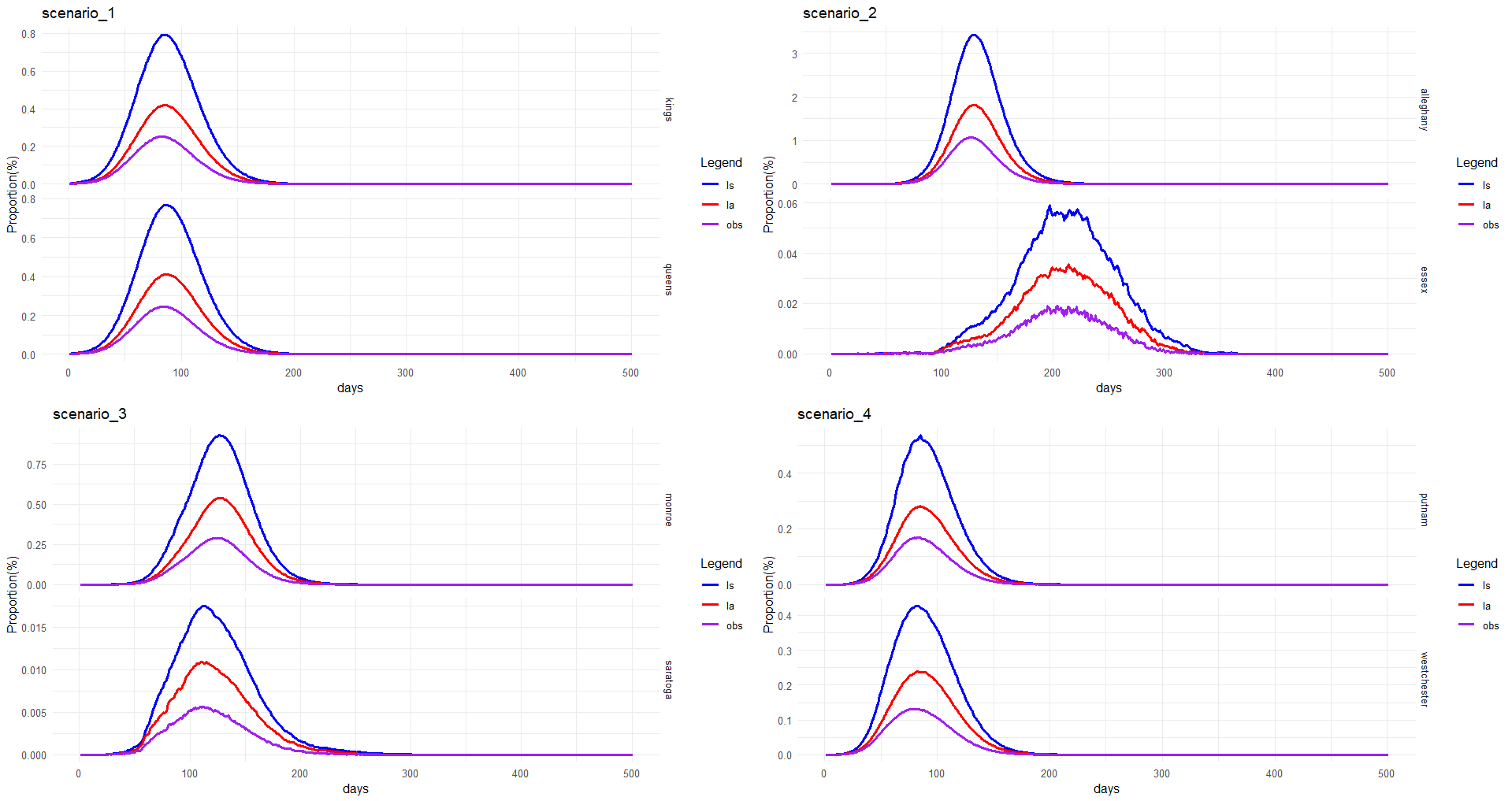


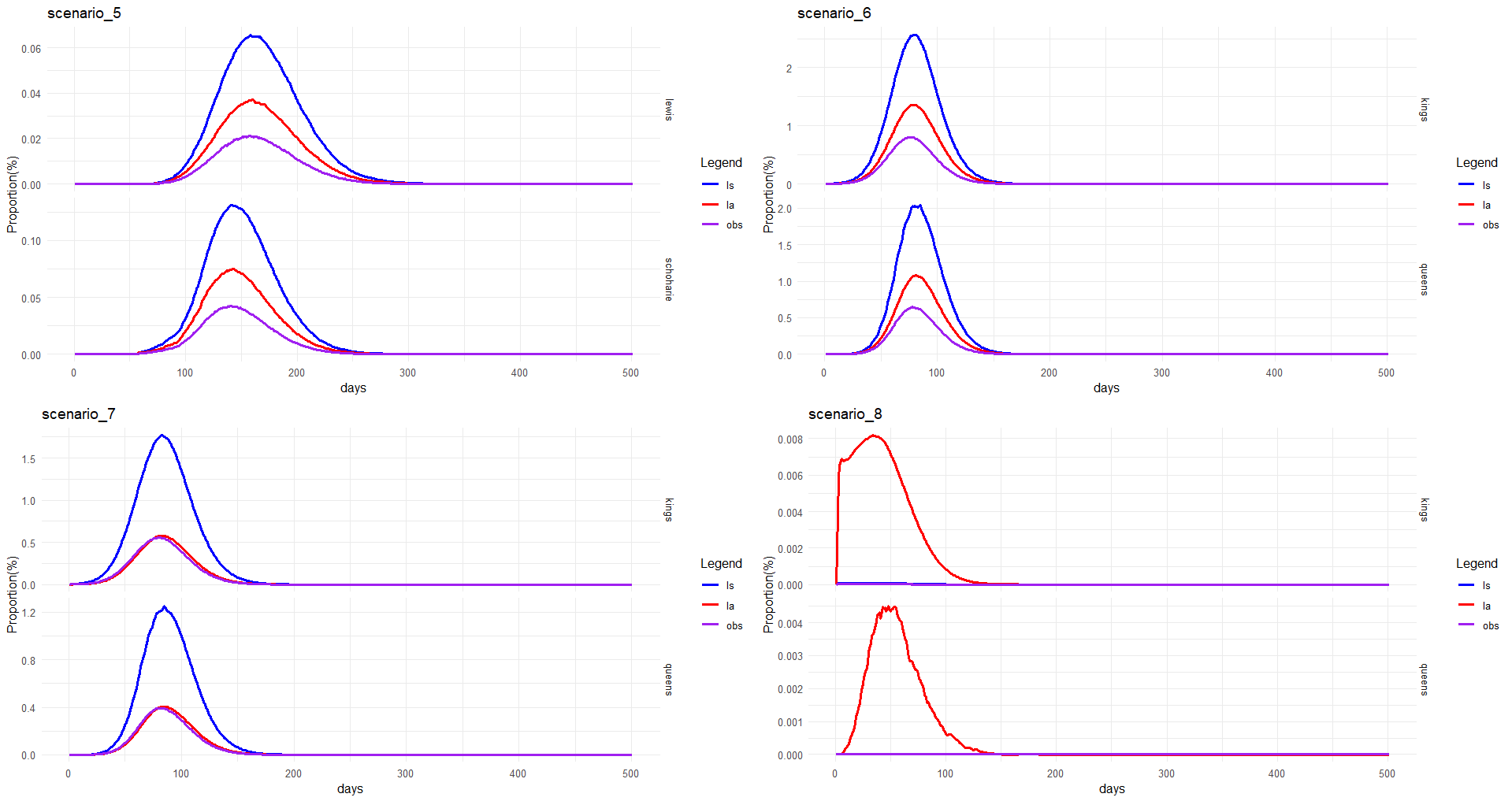


**Figure Captions**

1. S1 Fig: Simulated scenarios epidemiological curve embedding perturbed mobility matrices. *Scenarios 1 and 2 the disease is spread in two large and small counties, respectively; Scenarios 3, 4, and 5 the epidemic occurred in counties with no, medium to high connectivity with neighboring counties; Scenarios 6, 7, and 8 key parameters such as the burden of asymptomatic, the contact rate varied. 𝐼^𝑠^, 𝐼^𝑎^, and Obs are infected symptomatic, infected asymptomatic, and observed.*
