# Supplementary material for: A standardised differential privacy framework for epidemiological modeling with mobile phone data
Source: PLOS Digit Health. 2023 Oct 27;2(10):e0000233. doi: 10.1371/journal.pdig.0000233 (PMC10610440; doi:10.1371/journal.pdig.0000233)
Supplement: Attachment — Submitted filename: Point to point response to reviewers.pdf [file pdig.0000233.s002.pdf]

Point-by-point response to reviewers

We thank the reviewers for their insightful comments. We have provided a point-by-point response below and have incorporated their suggestions into the manuscript.

Response to reviewer #1

Reviewer #1: This paper addresses an important important topic, and presents a timely application of differential privacy. However, I believe that the presentation needs to be improved before publication.
Specifically, while I believe the privacy methodology to be sound, I have concerns about its presentation:

1. The exact mechanism used is never explicitly described, and as such it is hard to ensure that it satisfies differential privacy (especially in regards to the computation of the sensitivity). My understanding is that the original data already comes in aggregated form (transition matrices for 8-hour slots), to which individual users contribute at most once (sensitivity = 1). Noise is then added to these matrices before they are aggregated to form daily transition matrices. Is that accurate?

Yes, that is accurate. To protect the privacy of individuals who have provided this data we used a method that adds noise to already differentially private data. We used the mobility data from Camber System as our original data. We overlaid it with noise using a Laplace mechanism since the third-party data provider could not provide non-processed data. In light of this, we clarify how the synthetic data was obtained (method section) [lines 227-229].

2. In line 233, you mention that the data already has an "initial layer of privacy noise". This should be explained in more detail: what mechanism was used? Is it DP? If so, what privacy budget was used?

Due to the sensitive nature of mobility data and the fact that Camber System, our data provider does not disclose the detailed privacy protection implementations due to intellectual property reasons, we were not able to know the exact method used, which made it difficult for us to calculate the privacy budget as we discussed [Lines 245-246].

3. The computation of epsilon on line 265 is inaccurate, but I suspect this is due to a typo in the value of delta. It seems you used 1.75e-6, which is reasonable. It would also be good to explain briefly how you chose this value of delta.

Yes, the observation of the reviewer is accurate. We used the tight bound composition theorem where we arbitrarily chose $\epsilon = 0.01$ and $\delta = 2^{-30}$ and we found the optimal ϵ', δ' such that the composition of k mechanisms, each of which is (ϵ_0, δ_0)- differentially private, is (ϵ', δ')-differentially private as described by Murtagh and Vadhan (2016). [Line 276]

The choice of δ should be smaller than $1/n$, where n is the total number of people in the database. We choose $\delta = 2^{-30}$ because it is sufficiently small to satisfy this condition.

4. In lines 31 and 44, the paper mentions that an "epsilon of 0.05" could be used: this is a bit misleading due to the composition, and the total value (around 4 after advanced composition) should be used instead in these segments.

The reviewer raised an important point and we have clarified this a bit in our discussion. Previous studies have recommended much lower levels of noise to ensure unbiased estimates of epidemiological parameters. In our case,

we find that even with a method incorporating a minimal amount of noise into the system before our analysis, we can accept a much higher noise level than previously expected without severely biasing certain epidemiological parameters of interest. In this case, our value is at least an epsilon of 0.05. We have added this to our discussion on lines 217-222.

I found the epidemiological analysis interesting (although this is outside my area of expertise), but I think the results could be presented in a clearer way. At the moment, it is sometimes unclear whether the focus is on the results themselves or the impact of the privacy noise on the results. If possible, it would be good to show, e.g., a plot displaying how DP impacts key metrics. Your recommendation in the abstract of using epsilon=0.05 should also be more explicit in the results section.

Thank you for your comments, we have more directly commented on the epsilon threshold of interest in the results section on lines 125-128. This section now reads: As metrics can exist on very different scales we calculate the normalized distribution of bootstrapped metrics where a minimum amount of noise is added. We then compare this to the median value of bootstrapped values at increasing values of noise to describe the change from expectation.

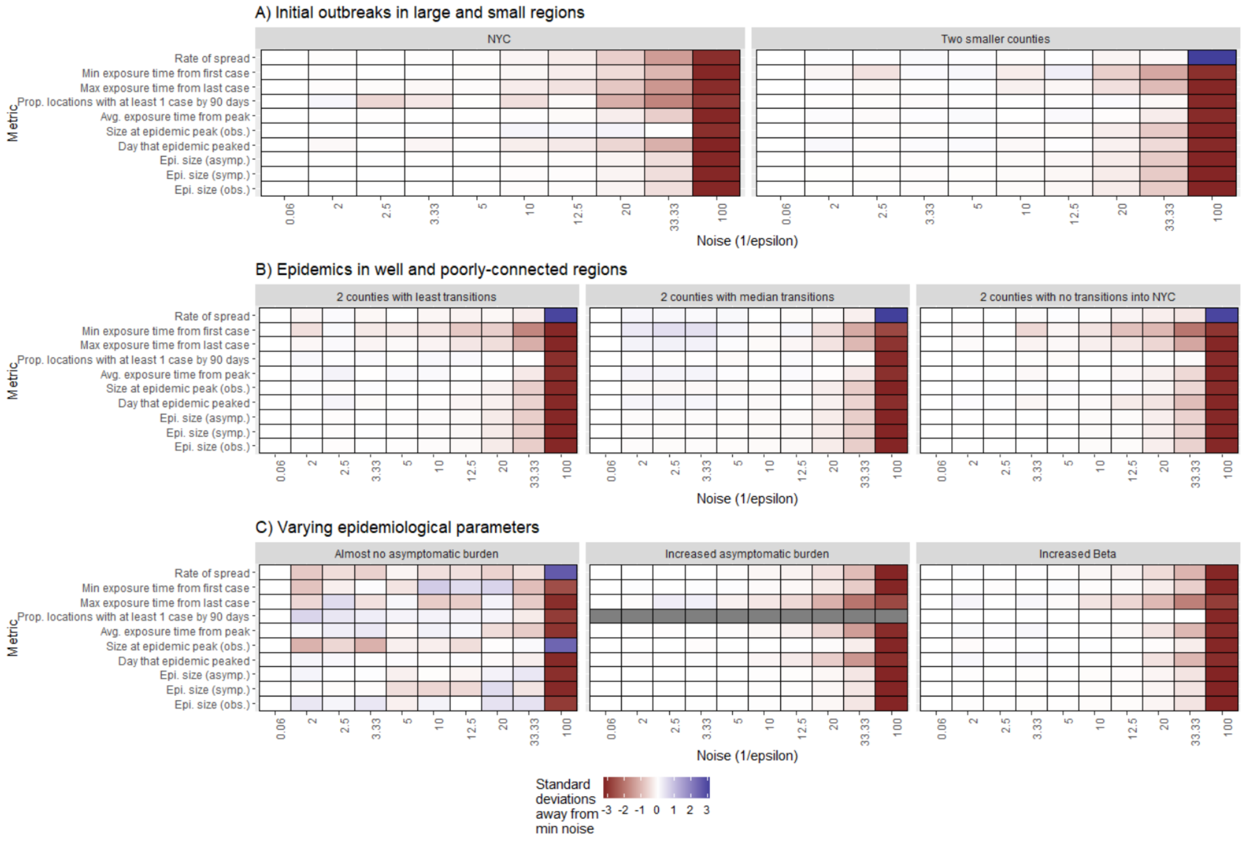

Minor comments:

- Could you please explain why and how you resample the data (line 238), and what impact (if any) it would have on the analysis?

Thank you for this question, we were primarily interested in the mobility network in New York State during an epidemic period. This time period included 90 days' worth of data, however, we wished to evaluate various types of

epidemics which in simulation can take more or less time. To extend this period we sampled randomly from our dataset conditioning for location and day of the week. As we resampled across simulations we would not expect any single location or time period to wash out the primary effects of the added noise on the stability of our epidemiological metrics of interest.

- Your definition of differential privacy (lines 244-245) should include delta, as you use it later for advanced composition.

Lines 251-261 we re-adjusted the definition of differential privacy taking into account the security parameter delta.

- I was a bit surprised by some of the references cited for the privacy side. In line 73, if you wish to say that aggregated data can be re-identified, I would cite https://arxiv.org/abs/1708.06145 rather than [8]. In line 75, I think references [9-15] do not represent "several privacy frameworks", at least as would be understood by the computer science community (i.e., k-anonymity, l-diversity, federated learning). Similarly, reference [18] on line 90 points to the OpenDP website, which does not support the claim. For that sentence, it would also be good to add references for the use of DP by Google etc. (see, e.g., https://desfontain.es/privacy/real-world-differential-privacy.html for a list of up-to-date references)

Thank you to the reviewer for bringing these references to our attention, and we have adjusted the references cited accordingly [lines 73, 75, 90].

Reviewer #2: The authors present an interesting paper on the impact of differential privacy techniques applied to mobility data on the output of epidemiological models. I believe this paper is very relevant. Given the central role that mobility data have acquired during the past years in computational epidemiology, similar contributions are needed as we build better data and modeling preparedness for the next global health emergency.
I do not have major concerns with the validity of the work, but I have some remarks mostly aimed at improving the accessibility of the paper:

1) It was not straightforward for me to understand what Fig 1 is showing and connect this with the commentary in the Results section. I would recommend to include a caption to Fig.1 and a brief description of it at the beginning of the results section. For example, apart from the legend in the figure it is never mentioned what the colors of the heatmap in Fig. 1 indicate and how they should be interpreted.

We have now added this on lines 125-128 and in the caption to the figure. The addition reads: As metrics can exist on very different scales we calculate the normalized distribution of bootstrapped metrics where a minimum amount of noise is added. We then compare this to the median value of bootstrapped values at increasing values of noise to describe the change from expectation.

2) The epidemiological metrics need a better explanation. Currently, they are just listed along with references in the methods section. While the interpretation of some metrics is quite self-explanatory, for others it may not be as straightforward especially for readers that are less familiar with epidemiological modeling. Therefore, I recommend to include more details on the definition of these metrics in the Methods section.

We thank the reviewer for this comment and have included details on the metrics [Lines 304-332], where we define each metric, show how it computed for our analyses and link to other epidemiological research which have sought to infer these metrics of interest.

3) I have similar remark also for the 8 epidemiological scenarios considered. At the current stage, the methods section has a very brief paragraph related to their description. I believe the paper would benefit from more details about the simulation scenarios.

We included a paragraph describing the rationale and relevance of the scenario as suggested by the reviewer [Lines 338-341].

“We explored several spatial epidemiological questions (Table 2) with our scenarios, including how the place of outbreak affects the dynamics of the disease, how connectivity networks could potentially affect epidemic dynamics, and how DP might ultimately affect the metric we are interested in. “

4) I would like to see clarified a bit more the sampling procedure of the mobility matrix. It is my understanding that the simulations last for 500 days, while the mobility data covers roughly 4 months. It is unclear to me at the moment how we go from a matrix M_t that varies daily for ~4 months to the 500 days one.

Thank you for this question, we were primarily interested in the mobility network in New York State during an epidemic period. This time period included 90 days' worth of data, however, we wished to evaluate various types of epidemics which in simulation can take more or less time. To extend this period we sampled randomly from our dataset conditioning for location and day of the week. As we resampled across simulations we would not expect any single location or time period to wash out the primary effects of the added noise on the stability of our epidemiological metrics of interest. [lines 245-248].

5) The providers of mobility data apply privacy filters to ensure that the privacy of users is preserved. Therefore, researchers receive preprocessed data that has undergone privacy-preserving algorithms. This is also the case with the data used in the paper under consideration. As the authors mentioned, the data has undergone privacy-preserving algorithms twice. However, I have two questions concerning this point.

5a) First, I wonder if this can bias the maximum estimated threshold of noise that can be applied without biasing too much the epidemiological metrics. My point is that it may be higher than 20 as the data have already some noise. While I do not think authors should change their analysis, this point should be at least discussed.

As the reviewers mentioned, the noise infused into the dataset prior to its acquisition could impair the precision of the epidemiological metrics. Previous studies have suggested a lower threshold of noise to be used to ensure unbiased estimates of epidemiological parameters of interest. In our research, we should that even with some minimal initial noise added, our key epidemiological parameters of interest were unbiased up to at least an epsilon of 0.05. We have addressed this in our discussion on lines ~~XXX~~. 198-205

5b) My second point concerns the authors' approach to operationalizing the proposed methodology. In its current form, the paper lacks a comprehensive discussion on this topic. Mobility data is typically provided with noise, and researchers have limited knowledge about the methods used by data providers to ensure user privacy. Therefore, it is unclear how the proposed methodologies can overcome these challenges. One potential solution is to collaborate with data providers and encourage them to apply privacy-preserving algorithms that do not compromise the accuracy of epidemic models. Alternatively, privacy-preserving algorithms can be reapplied to the data, but this approach requires more caution as different providers may have applied vastly different preprocessing techniques. Overall, the paper would benefit from a more in-depth exploration of the potential challenges associated with operationalizing the proposed methodology and a thorough discussion of possible solutions.

Thank you for this comment, we have addressed this with a section in our discussion on our intended use of the results of this study. Specifically on lines 217-222, we have stated that: “Data providers can interact with researchers in many different ways and the goal of this study is not to systematize this relationship. Instead, this “plug-and-play” framework can be used by researchers to simulate the effects of the application of differential privacy methods on their epidemiological parameters of interest. This would allow researchers to have an informed discussion with data providers before the data are sourced to identify an optimal threshold of noise which protects user privacy while also allowing for unbiased estimates of epidemiological parameters to be inferred.”

We hope that this paper can be used as guidance by researchers who work closely with data providers to ensure that epidemiological questions they want to ask are responded to appropriately. In cases where researchers are unable to work directly with data providers, researchers could use the provided method to test the limitations of their results depending on their epidemiological metric of interest
